# Behavioral and Neurotransmitter Abnormalities in Mice Deficient for Parkin, DJ-1 and Superoxide Dismutase

**DOI:** 10.1371/journal.pone.0084894

**Published:** 2013-12-26

**Authors:** Meghan R. Hennis, Katherine W. Seamans, Marian A. Marvin, Bradford H. Casey, Matthew S. Goldberg

**Affiliations:** 1 Department of Neurology and Neurotherapeutics, University of Texas Southwestern Medical Center, Dallas, Texas, United States of America; 2 Department of Psychiatry, University of Texas Southwestern Medical Center, Dallas, Texas, United States of America; Ecole Polytechnique Federale de Lausanne (EPFL), Switzerland

## Abstract

Parkinson’s disease (PD) is a progressive neurodegenerative disease characterized by loss of neurons in the substantia nigra that project to the striatum and release dopamine. The cause of PD remains uncertain, however, evidence implicates mitochondrial dysfunction and oxidative stress. Although most cases of PD are sporadic, 5-10% of cases are caused by inherited mutations. Loss-of-function mutations in *Parkin* and *DJ-1* were the first to be linked to recessively inherited Parkinsonism. Surprisingly, mice bearing similar loss-of-function mutations in *Parkin* and *DJ-1* do not show age-dependent loss of nigral dopaminergic neurons or depletion of dopamine in the striatum. Although the normal cellular functions of Parkin and DJ-1 are not fully understood, we hypothesized that loss-of-function mutations in Parkin and DJ-1 render cells more sensitive to mitochondrial dysfunction and oxidative stress. To test this hypothesis, we crossed mice deficient for Parkin and DJ-1 with mice deficient for the mitochondrial antioxidant protein Mn-superoxide dismutase (SOD2) or the cytosolic antioxidant protein Cu-Zn-superoxide dismutase (SOD1). Aged *Parkin* ^*-/-*^ *DJ-1* ^*-/-*^ and Mn-superoxide dismutase triple deficient mice have enhanced performance on the rotorod behavior test. Cu/Zn-superoxide dismutase triple deficient mice have elevated levels of dopamine in the striatum in the absence of nigral cell loss. Our studies demonstrate that on a *Parkin/DJ-1* null background, mice that are also deficient for major antioxidant proteins do not have progressive loss of dopaminergic neurons but have behavioral and striatal dopamine abnormalities.

## Introduction

Parkinson’s disease (PD) is the second most common neurodegenerative disorder after Alzheimer’s disease and afflicts millions of people worldwide. The primary clinical symptoms are bradykinesia, resting tremor, rigidity, and postural instability. These symptoms are caused by the loss of dopaminergic innervation of the striatum and increase in severity over time due to selective, progressive nigral dopaminergic neuron loss. Most cases of PD are sporadic and the underlying cause of neuronal death remains unknown. The greatest risk factor for PD is age. About 5 to 10% of all cases are caused by inherited mutations [1-6]. Loss-of-function mutations in the *Parkin* and *DJ-1* genes were the first mutations to be causally linked to recessive parkinsonism [7,8]. Both genes are widely expressed throughout the brain and other tissues [9-13]. The mechanism by which loss of Parkin or DJ-1 function causes parkinsonism remains unclear.

Mice with targeted disruption of *Parkin* or *DJ-1* genes do not show robust neuropathology or age-dependent symptoms related to PD, suggesting the existence of compensatory mechanisms that may protect mice from the neurodegeneration and consequent motor symptoms that occur in humans with Parkin or DJ-1 mutations [14-33]. However, *Parkin* knockout and *DJ-1* knockout mice are more susceptible to PD-related neurodegeneration induced by various stresses including exposure to neurotoxins or to lipopolysaccharide (LPS) [18,22,24,34].

Overexpression of Parkin or DJ-1 is neuroprotective both *in vitro* and *in vivo* [34-40]. Parkin has been found to function as an E3 ubiquitin ligase [41] and is known to promote autophagy of dysfunctional mitochondria [42], which are major cellular sources of free radicals and oxidative stress. Mutations in *Parkin* result in impaired mitochondrial respiration and increased markers of oxidative stress [25,43-48]. The exact cellular function of DJ-1 remains uncertain, but it has been reported to be an atypical peroxiredoxin-like peroxidase [14] and may be a sensor of oxidative stress [49]. Cysteine 106 of DJ-1 is required for neuroprotection and is crucial for DJ-1 to localize to mitochondria under stress conditions [22,37,50-53].

Together, these data suggest that oxidative damage may be an important factor in the development of PD caused by *Parkin* and *DJ-1* mutations. Oxidative stress has been implicated as a potential cause of idiopathic PD because postmortem examinations of PD patients show increased oxidative damage in neurons [54-57]. Furthermore, the capacity of cells to clear reactive oxygen species and repair oxidative damage to proteins, lipids and nucleic acids diminishes with age [58].

Two superoxide dismutase proteins, cytoplasmic Cu/Zn-superoxide dismutase (SOD1) and mitochondrial Mn-superoxide dismutase (SOD2) are the among the most abundant antioxidant proteins in the brain and are important for protecting neurons from oxidative stress. The superoxide dismutase proteins remove toxic superoxide by catalytically converting it to oxygen and hydrogen peroxide. Some studies have suggested that abnormalities in SOD1 or SOD2 may contribute to the development of PD [59-61], although no mutations in *SOD1* or *SOD2* have been causally linked to PD. In flies, expression of human SOD1 is protective against neuronal loss due to inactivation of PINK1, another gene linked to recessive parkinsonism [62]. In mice, overexpression of SOD2 is protective against nigral dopamine neuron loss induced by the neurotoxin 6-hydroxydopamine while partial SOD2-deficiency increases 1-methyl-4-phenyl-1,2,3,6-tetrahydropyridine (MPTP) sensitivity [63,64]. Levels of SOD1 mRNA and SOD1 activity are significantly reduced in PD patients [65-67]. Overexpression of SOD1 or SOD2 *in vitro* and *in vivo* is protective against MPTP, methamphetamine and 6-hydroxydopamine toxicity [64,66,68-71]. MPTP exposure increases the protein levels of both SOD1 and SOD2 [72], suggesting that these proteins are important for mitigating oxidative stress in response to toxins. Together, these studies indicate that SOD1 and SOD2 protein or activity levels may be key determinants of susceptibility to nigral cell loss in mice.

Aged *DJ-1* ^*-/-*^ mice have increased SOD2 in brain mitochondria, suggesting that up-regulation of antioxidant proteins may prevent PD-linked loss-of-function mutations from causing nigral cell loss in mice [14]. We hypothesized that eliminating potential compensatory upregulation of superoxide dismutase activity would result in PD-related neurodegeneration in *Parkin* and *DJ-1* knockout mice. To test this hypothesis and to potentially generate better PD animal models, we crossed mice deficient for Parkin and DJ-1 with mice deficient for SOD1 to generate triple mutant mice deficient for all three genes (Parkin^-/-^DJ-1^-/-^SOD1^-/-^). We also generated Parkin^-/-^DJ-1^-/-^SOD2^+/-^ mice by crossing Parkin^-/-^DJ-1^-/-^ mice with heterozygous SOD2 knockout mice because homozygous SOD2 deficiency is perinatal lethal [73] while heterozygous SOD2 knockout mice have approximately 50% decreased SOD2 activity and increased oxidative damage [74,75]. The triple mutant mice did not exhibit age-dependent nigral neuron loss, dopamine depletion, or motor behaviors characteristic of PD. Contrary to our hypothesis, Parkin^-/-^DJ-1^-/-^SOD1^-/-^ triple mutant mice had increased striatal dopamine levels and Parkin^-/-^DJ-1^-/-^ mice showed improved rotarod performance. These results are consistent with our previous study showing improved rotarod performance in *Parkin* ^*-/-*^ *DJ-1* ^*-/-*^ mice and increased striatal dopamine in *Parkin* ^*-/-*^ *DJ-1* ^*-/-*^ *Gpx1* ^*-/-*^ mice [76] and consistent with the increased striatal dopamine observed in aged *Parkin* ^*-/-*^ *DJ-1* ^*-/-*^ *PINK1* ^*-/-*^ mice [23]. This study demonstrates that superoxide dismutase is not critical for preventing nigral cell loss caused by Parkin and DJ-1 deficiencies. The increase in striatal dopamine in Parkin^-/-^DJ-1^-/-^SOD1^-/-^ mice indicates functional abnormalities within the nigrostriatal pathway. These behavioral and neurotransmitter abnormalities suggest that the pathogenic mechanisms of *Parkin* and *DJ-1* mutations may involve dysregulation of nigrostriatal dopaminergic neurotransmission.

## Materials and Methods

### Ethics Statement

Experimental procedures involving the use of animals or animal tissue were performed in accordance with the NIH Guidelines for Animal Care and Use and approved by the Institutional Animal Care and Use Committee at The University of Texas Southwestern Medical Center.

### Animals

Parkin knockout mice and DJ-1 knockout mice were generated as previously described [19,20] and backcrossed to strain C57BL/6J for 10 generations, then intercrossed for two generations to obtain homozygous double knockout mice (Parkin^-/-^DJ-1^-/-^) and wild type controls. *SOD1* and *SOD2* knockout mice on a C57BL/6 background were kindly provided by Dr. Holly VanRemmen with permission from Dr. Ting-Ting Huang [73,77]. *SOD1* and *SOD2* knockout mice were crossed with Parkin, DJ-1 double knockout mice for two generations to produce Parkin^-/-^DJ-1^-/-^SOD1^+/-^, Parkin^-/-^DJ-1^-/-^SOD1^-/-^ and Parkin^-/-^DJ-1^-/-^SOD2^+/-^ mice. When possible, paired littermates were used as controls; when not possible, wild type mice from the same line to which the mutant mice were backcrossed were used as controls (C57BL/6). Animals were housed in a climate-controlled facility with ventilated cages and standard commercial lab diet. Behavioral tests were performed between 10 AM and 4 PM during the 6 AM to 6 PM light cycle.

### Behavioral tests

#### Rotarod

To measure motor coordination and skill, mice were placed on an accelerating rotarod (IITC Life Science Inc) and the speed of rotation was increased from 5 to 45 revolutions per minute (RPM) over 5 minutes. The latency to fall from the rotarod was recorded. Data were collected for 4 trials per day for 2 days.

#### Locomotor

To measure spontaneous locomotor activity, mice were placed individually in a clean cage within an infrared photobeam activity monitor (San Diego Instruments) and were allowed to move freely in the dark for 2 hours. The number of beam breaks was recorded in 5-minute bins as a measure of locomotor activity.

#### Open Field Activity

Mice were placed in the periphery of a novel open field environment (44 cm x 44 cm, walls 30 cm high) and allowed to explore for five minutes. The animals were monitored from above by a video camera connected to a computer running video tracking software (Ethovision 3.0, Noldus, Leesburg, Virginia) to determine the time, distance moved and number of entries into two areas: the periphery (5 cm from the walls) and the center (14 cm x 14 cm). The open field arenas were wiped and allowed to dry between mice. 

#### Elevated Plus Maze

Mice were placed in the center of a black Plexiglas elevated plus maze (each arm 30 cm long and 5 cm wide with two opposite arms closed by 25 cm high walls) elevated 31 cm from the floor and allowed to explore for five minutes. The animals were monitored from above by a video camera connected to a computer running video tracking software (Ethovision 3.0, Noldus, Leesburg, Virginia) to determine time spent in the open and closed arms, time spent in the middle, and the number of entries into the open and closed arm. The apparatus was wiped and allowed to dry between mice. 

#### Dark-Light Activity

Mice were placed into a black Plexiglas chamber (25 cm x 26 cm) and allowed to explore for two minutes. After the habituation period, a small door was opened allowing them to access the light side of the apparatus (25 cm x 26 cm lit to approximately 1700 lux) for ten minutes. The animals were monitored by seven photobeams in the dark compartment and eight photobeams on the light side connected to a computer which recorded the time spent in each compartment, latency to enter the light side and the number of entrances to each compartment (Med-PC IV, Med Associates, St. Albans, VT). The dark-light apparatus was wiped and allowed to dry between mice. 

#### Acoustic Startle Response

Acoustic startle response behavior was measured using a San Diego Instruments SR-Lab Startle Response System (San Diego, CA). Mice were placed into Plexiglas holders and allowed to acclimate to the chamber and background white noise (70 dB) for five minutes. After acclimation, startle stimuli (80, 90, 100, 110 and 120 dB, 40 ms, white noise) were presented in pseudorandom order with an average interstimulus interval of 20 seconds (range 13 - 27 seconds). Each startle response was computer recorded by force transducers. The Plexiglas holders were wiped and allowed to dry between mice.

#### Forced Swim Test

Mice were placed in a 16.5 cm diameter beaker of water (21-25° C) filled to a depth of seven inches. The mice remained in the water for 6 minutes and were then removed and allowed to dry in a clean dry cage before returning to their home cage. The water was changed between each subject. The mice were monitored from the side by camera and video images were recorded for later analysis. The last four minutes of the test were scored for latency to the first immobility and total time spent immobile. The experimenter scoring the behavior was blind to the genotypes. Immobility was defined as no body or limb movement other than a minimal forelimb movement required for keeping the head above water.

#### Grip strength

Mice were suspended by the tail and allowed to clasp a grip bar with both forelimbs. Gentle constant horizontal resistance was applied until the mouse released the bar. Force was measured in grams by a force transducer attached to the bar. The greatest three of five trials per mouse were averaged. 

### Stereology of Dopaminergic Neurons

Brains were removed and placed in 10% neutral buffered formalin at 4° overnight, processed for paraffin embedding and sectioned in the coronal plane at 20 micron thickness. Every fifth slide was stained and used for unbiased stereology. Slides were deparaffinized and rehydrated and blocked with 5% normal goat serum for 1 hour prior to incubation in primary antibody (anti-tyrosine hydroxylase AB152, Chemicon) diluted 1:1000 at 4°C overnight. Sections were washed, incubated with biotinylated goat anti-rabbit secondary antibody, horseradish peroxidase conjugated avidin (ABC Elite, Vector) and were developed in DAB solution with NiCl enhancement prior to dehydration and coverslipping. A microscope with a motorized stage and Stereoinvestigator software was used to count the tyrosine hydroxylase positive neurons in the substantia nigra of each section, with a counting frame of 50 microns by 50 microns and a grid size of 100 microns by 100 microns. The total number of bilateral substantia nigra dopamine neurons was estimated by counting neurons from one hemisphere.

### Measurement of tissue neurotransmitter levels

Mice were euthanized and the striatum was quickly dissected on an ice-cold glass dish, weighed, frozen on dry ice and stored at -80 prior to analysis. Samples were combined with 50-fold (weight:volume) ice-cold 0.1 N perchloric acid containing 0.2 mM sodium metabisulfite. The tissue was disrupted by brief sonication and centrifuged at 4°C for 20 minutes at 15,000 x g to pellet proteins and cell debris. 200 µL of the supernatant was transferred to a clean tube, and 20 µL was injected onto an HPLC with a C18 column and eluted with isocratic MDTM mobile phase (ESA) at a rate of 0.6 mL/min. Monoamines were detected with a model 5014B electrochemical cell (ESA) set to a potential of +220 mV. Peak areas were normalized to tissue weight and compared to external standards for quantification.

### Tritiated Dopamine Ligand Binding to D1 and D2-like Receptors in the Striatum

The rostral half of unfixed mouse brains were sectioned in the coronal plane on a cryostat at a thickness of 20 microns. Slides were preincubated in assay buffer (50 mM Tris, 120 mM sodium chloride, 5 mM potassium chloride, 1 mM magnesium chloride, 40 nM ketanserin) for 20 min at room temperature. Slides were then incubated in buffer containing either 2 nM tritiated SCH 23390 (PerkinElmer, Boston, MA) or 5 nM tritiated spiperone (PerkinElmer, Boston, MA). Cold competition of tritiated siperone with 10μm cold siperone, or of tritiated SCH 23390 with 1μm cold SCH 23390 was used to assess non-specific signal. Following a 1 hour incubation, slides were washed twice in ice-cold buffer and then rinsed in cold water prior to incubation overnight at 4C in a desiccator with paraformaldehyde powder in the bottom to fix the tissue without washing away the ligand. Slides were dried for 2 hours in a desiccator with Dri-Rite and exposed to Kodak BioMax MS film with the Kodak BioMax TranScreen-LE Intensifying Screen for seven days (D1) or 5-7 weeks (D2). Films were analyzed for both density and area of binding using Adobe Photoshop software. Measurements from twenty sections per animal were averaged prior to statistical analysis 4 mice per genotype were included in the analysis. 

### Hematoxylin and eosin (H&E) staining

Muscle tissue was taken from the extensor digitorum longus (EDL) and soleus (SOL) muscles and frozen sections were incubated 45 minutes in hematoxylin stain followed by a water rinse, then a 0.3% hydrochloric acid in ethanol rinse. Slides were incubated in eosin for 2 minutes, dehydrated and mounted for imaging with a light microscope, as above. 

### Muscle fiber typing

Extensor digitorum longus (EDL) and soleus (SOL) muscles were dissected from wild type and *Parkin* ^*-/-*^ *DJ-1* ^*-/-*^ mice and flash frozen. Muscle were cut into thin sections and maintained at -20°C. Sections were stained with Metachromatic ATPase as previously published (Ogilvie 1990). Briefly, sections were pre-incubated with ATPase 8 minutes (pH 4.5), rinsed twice for 3 minutes in Tris buffer (pH 7.8), then incubated with ATP (pH9.4) for 25 minutes at room temperature. Slides were rinsed 3 times with calcium chloride and counterstained with Toluene blue 0.1% for 1 minute, cleared in ethanol, then xylene, and mounted for imaging with a light microscope, as above. 

### Statistical Analysis

All analysis was performed using SigmaPlot. All experiments were analyzed by ANOVA to account for multiple comparisons. Two way repeated measures ANOVA was performed where appropriate to account for multiple samplings over time. Pairwise and posthoc comparisons were performed by Tukey test, unless otherwise noted. Student’s t-test was used in lieu of ANOVA where appropriate. Results are presented as mean ±standard error of the mean.

## Results

### No neuronal loss in the substantia nigra of mutant mice

**Figure 1:**
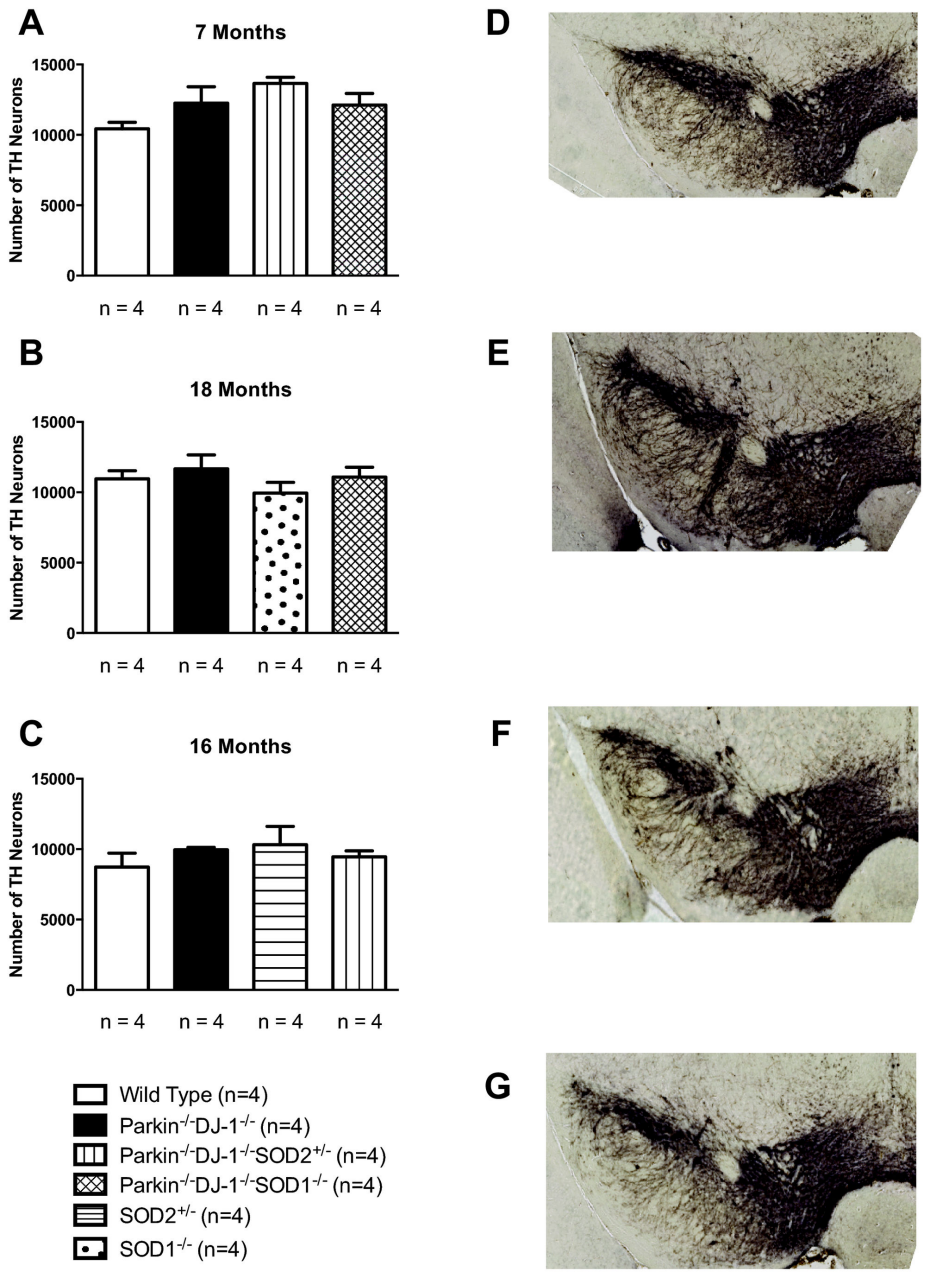
No changes in the total bilateral number of TH-positive nigral neurons estimated by rigorous stereology. Dopaminergic neurons of separate cohorts of mice were counted for 7 month old mice (A) and 18 month old mice (B) and 16 month old mice (C). n = 4 for all genotypes. One-way ANOVA showed no differences between any of the genotypes (p ≥ 0.1). Representative images of TH-stained coronal sections used for analysis are shown for 7 month old wild-type (D), Parkin^-/-^DJ-1^-/-^ (E), Parkin^-/-^DJ-1^-/-^SOD2^+/-^ (F) and Parkin^-/-^DJ-1^-/-^SOD1^-/-^ (G).

Triple knockout mice bearing combined loss-of-function mutations in the PD-linked genes *Parkin* and *DJ-1*, as well as the antioxidants *SOD1 or SOD2*, were born at the expected Mendelian ratio and had no apparent differences in viability compared to wild type mice. Because the pathological hallmark of PD is the progressive loss of dopamine neurons in the substantia nigra and previously published data indicate that *Parkin* ^*-/-*^ and *DJ-1* ^*-/-*^ mice do not have changes in numbers of nigral dopaminergic neurons [14,16,18-24,28,33], we postulated that mice with a deficiency for major antioxidant proteins in addition to Parkin and DJ-1 would show an age-dependent loss of dopamine neurons in the substantia nigra and thereby model human PD neuropathology. We used rigorous stereology to obtain unbiased estimates of the number of dopaminergic neurons, marked by immunohistochemical staining for tyrosine hydroxylase (TH), in coronal paraffin sections of wild type and mutant mice. We analyzed cohorts of mice at ages 7, 16 and 18 months. There was no statistically significant difference between the wild type mice and any of the mutant mice at any age by ANOVA (Figure 1), indicating no change in the number of nigral dopaminergic neurons in *Parkin* ^*-/-*^ *DJ-1* ^*-/-*^ *SOD1* ^*-/-*^ mice or *Parkin* ^*-/-*^ *DJ-1* ^*-/-*^ *SOD2* ^*+/-*^ mice compared to wild type mice*.*

### Dopamine levels are elevated in the striatum of triple mutant mice

**Figure 2:**
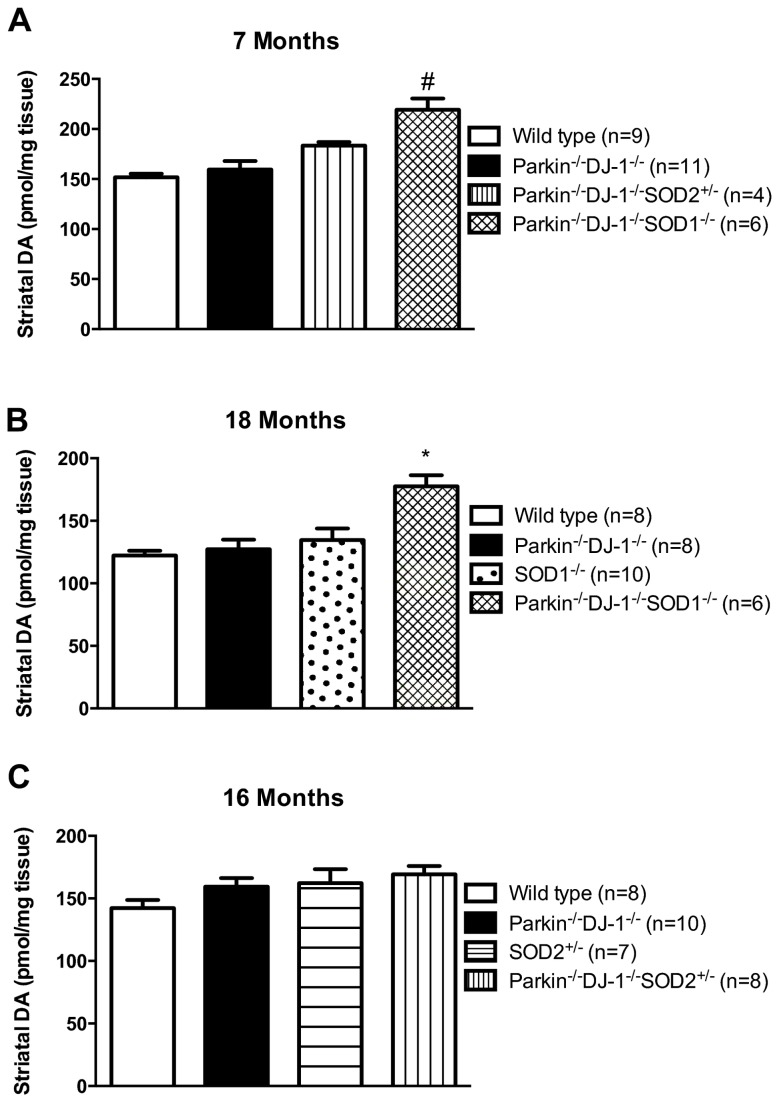
Dopamine levels are elevated in Parkin^-/-^DJ-1^-/-^SOD1^-/-^ mice. Total dopamine levels were measured by HPLC analysis in separate cohorts of mice ages 7 (A), 18 (B) and 16 (C) months (n ≥ 6). (*p ≤ 0.001 by one way ANOVA; #p<0.001 by Kruskal-Wallis one way ANOVA on ranks).

Although we observed no change in dopaminergic nigral neurons, loss of dopaminergic terminals in the striatum may precede loss of cell bodies in the substantia nigra. Therefore, we used HPLC with electrochemical detection to measure levels of dopamine, serotonin and their metabolites in the striatum of wild type and mutant mice. Our previous studies showed no changes in the level of striatal dopamine in *Parkin* ^*-/-*^ mice or *DJ-1* ^*-/-*^ mice compared to wild type mice [19,20]. Remarkably, we found a consistent and statistically significant increase in dopamine in the striatum of *Parkin* ^*-/-*^ *DJ-1* ^*-/-*^ *SOD1* ^*-/-*^ mice, but not in *Parkin* ^*-/-*^ *DJ-1* ^*-/-*^ *SOD2* ^*+/-*^ mice, compared to wild type mice (Figure 2) and no differences in the levels of dopamine or serotonin between wild type and *Parkin* ^*-/-*^ *DJ-1* ^*-/-*^ mice (Table 1).

**Table 1:** Striatum levels of dopamine, serotonin and metabolites in wild type and mutant mice.

**Age (months)**	**Genotype**	**DA**	**DOPAC**	**HVA**	**3-MT**	**5-HT**	**5-HIAA**	**DA turnover**	**5-HT turnover**
**7**	Wild type	139.6 ± 3.7	9.89 ± 0.65	9.97 ± 0.37	6.93 ± 0.32	5.05 ± 0.16	3.24 ± 0.11	0.19 ± 0.01	0.59 ± 0.02
**7**	Parkin^-/-^DJ-1^-/-^	147.0 ± 8.6	8.28 ± 1.03	10.03 ± 0.43	7.67 ± 0.48	5.00 ± 0.17	3.35 ± 0.15	0.18 ± 0.01	0.68 ± 0.03
**7**	Parkin^-/-^DJ-1^-/-^SOD1^-/-^	**202.2 ± 3.5**	10.20 ± 0.71	11.93 ± 0.65	7.09 ± 0.54	5.58 ± 0.13	3.98 ± 0.22	**0.14 ± 0.01**	0.65 ± 0.06
**7**	Parkin^-/-^DJ-1^-/-^SOD2^+/-^	169.1 ±11.1	9.12 ± 0.58	9.96 ± 0.12	8.08 ± 0.32	4.81 ± 0.10	3.08 ± 0.30	0.16 ± 0.01	0.58 ± 0.04
**16**	Wild type	142.2 ±6.6	7.98 ± 0.45	10.86 ± 0.52	7.65 ± 0.41	4.49 ± 0.21	3.13 ± 0.21	0.19 ± 0.01	0.73 ± 0.04
**16**	SOD2^+/-^	162.3 ±11.1	8.80 ± 0.69	12.92 ± 0.87	9.30 ± 0.49	4.50 ± 0.31	2.91 ± 0.21	0.19 ± 0.01	0.65 ± 0.02
**16**	Parkin^-/-^DJ-1^-/-^	159.4 6.9	8.37 ± 0.31	**13.73 ± 0.44**	**9.40 ± 0.30**	5.46 ± 0.30	3.50 ± 0.19	0.20 ± 0.004	0.64 ± 0.03
**16**	Parkin^-/-^DJ-1^-/-^SOD2^+/-^	169.3 ±6.6	10.35 ± 0.77	**14.23 ± 0.50**	**9.23 ± 0.56**	**5.19 ± 0.13**	3.44 ± 0.16	0.21 ± 0.01	0.72 ± 0.02
**18**	Wild type	122.3 ± 3.7	6.52 ± 0.25	8.13 ± 0.22	6.16 ± 0.34	4.17 ± 0.17	2.35 ± 0.12	0.17 ± 0.004	0.57 ± 0.03
**18**	SOD1^-/-^	134.5 ± 9.5	**8.00 ± 0.20**	**9.65 ± 0.28**	6.52 ± 0.42	4.60 ± 0.19	**3.01 ± 0.17**	0.19 ± 0.01	0.66 ± 0.03
**18**	Parkin^-/-^DJ-1^-/-^	127.3 ± 7.6	7.32 ± 0.43	8.78 ± 0.26	6.24 ± 0.13	4.79 ± 0.21	2.53 ± 0.13	0.18 ± 0.01	0.53 ± 0.02
**18**	Parkin^-/-^DJ-1^-/-^SOD1^-/-^	**177.6 ±8.9**	**8.97 ± 0.73**	**11.34 ± 0.61**	7.22 ± 0.24	**5.52 ± 0.19**	**3.49 ± 0.17**	0.16 ± 0.01	0.64 ± 0.04

Levels of dopamine (DA), DOPAC, HVA, 3-MT, serotonin (5-HT), 5-HIAA and turnover of DA and 5-HT in wild type and mutant mice at ages 7, 16 and 18 months are shown as mean ± SEM. Levels that were found to be significant by ANOVA (p<0.05) are shown in bold. DA turnover is calculated as (DOPAC + HVA +3-MT)/DA. Serotonin turnover is calculated as 5-HIAA/5-HT.

At ages 7 and 18 months, striatal levels of dopamine (Figure 2A, C) in *Parkin* ^*-/-*^ *DJ-1* ^*-/-*^ *SOD1* ^*-/-*^ mice were significantly higher compared to wild type mice (p<0.05). At age 7 months, the mice also had significantly increased dopamine turnover compared to wild type mice (p<0.05, Table 1). At age 18 months, serotonin (5-HT) and the dopamine metabolites 3,4-Dihydroxyphenylacetic acid (DOPAC) and homovanillic acid (HVA) in the striatum of *Parkin* ^*-/-*^ *DJ-1* ^*-/-*^ *SOD1* ^*-/-*^ mice were significantly higher compared to wild type mice (p<0.05, Table 1). Additionally, aged *SOD1* ^*-/-*^ mice have increased DOPAC, HVA and the serotonin metabolite 5-Hydroxyindoleacetic acid (5-HIAA) (p<0.05, Table 1).

Analysis of *Parkin* ^*-/-*^ *DJ-1* ^*-/-*^ *SOD2* ^*+/-*^ mice at 7 and 16 months showed a trend toward an increase in striatal dopamine in *Parkin* ^*-/-*^ *DJ-1* ^*-/-*^ *SOD2* ^*+/-*^ mice compared to wild type mice (p=0.101) (Figure 2C). Striatal serotonin, HVA and 3-methoxytyramine (3-MT) levels were significantly increased in aged *Parkin* ^*-/-*^ *DJ-1* ^*-/-*^ *SOD2* ^*+/-*^ mice compared to wild type mice (p<0.05, Table 1).

Separately, we examined TH immunoreactivity in the striatum of wild type, *Parkin* ^*-/-*^ *DJ-1* ^*-/-*^, *Parkin* ^*-/-*^ *DJ-1* ^*-/-*^ *SOD1* ^*-/-*^ and *Parkin* ^*-/-*^ *DJ-1* ^*-/-*^ *SOD2* ^*+/-*^ mice to see whether the increased striatal dopamine was accompanied by increased TH levels. Images of striata stained with DAB to measure TH-immunoreactivity were analyzed by densitometry using Image J software. In agreement with previous data [28], we found no differences in the intensity of TH immunostaining between wild type, *Parkin* ^*-/-*^ *DJ-1* ^*-/-*^, *Parkin* ^*-/-*^ *DJ-1* ^*-/-*^ *SOD1* ^*-/-*^ or *Parkin* ^*-/-*^ *DJ-1* ^*-/-*^ *SOD2* ^*+/-*^mice (p>0.5) (data not shown).

### D1 and D2-like receptor densities in the striatum of mutant mice are similar to wild type

**Figure 3:**
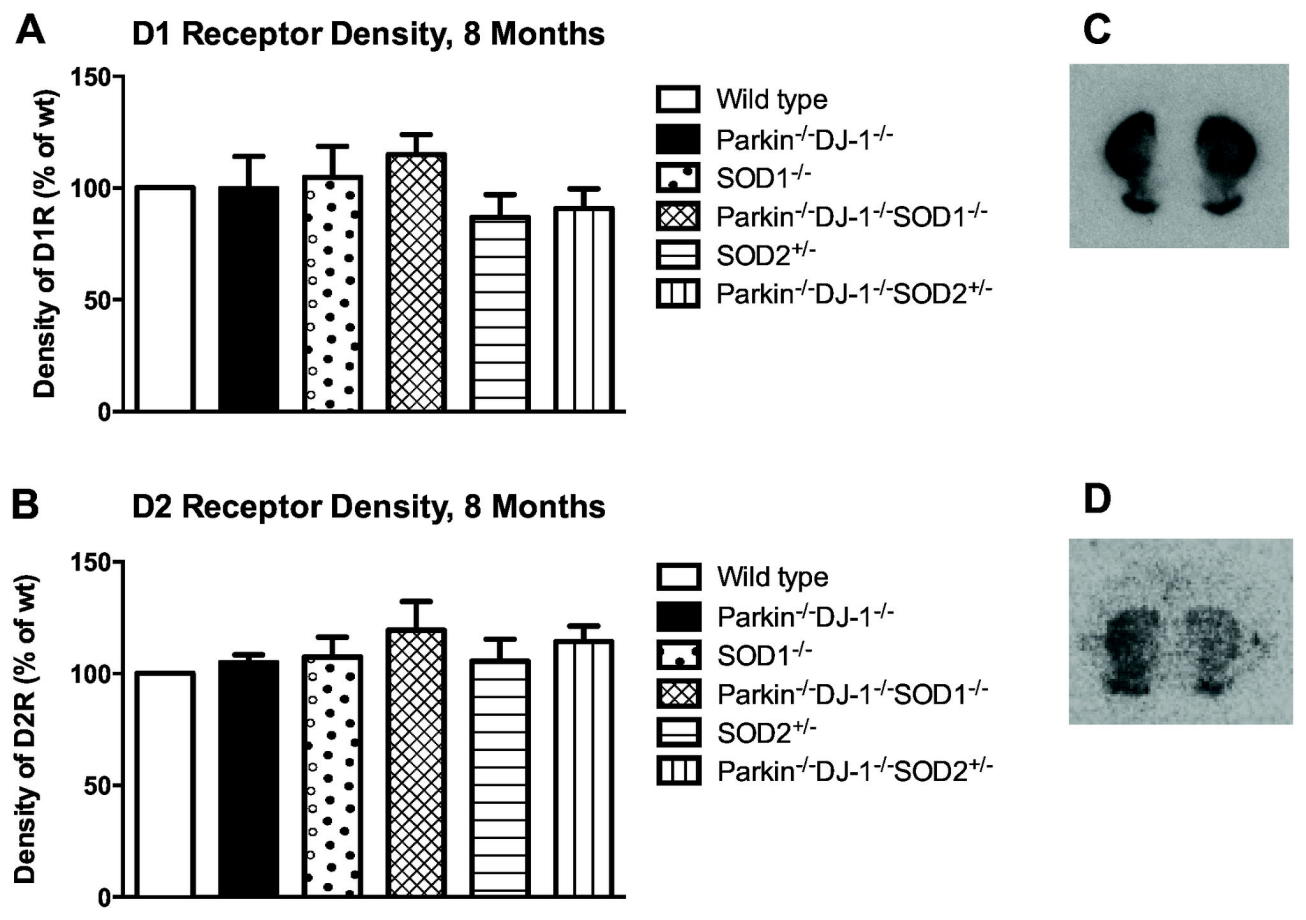
Density of D1 and D2 receptors is unchanged in mutant mice. (A-B) Striatal D1 and D2 dopamine receptor density was measured by radioligand binding to coronal sections through the striatum (n ≥ 3 animals per genotype). One-way ANOVA showed no differences between genotypes in the density of D1 or D2-type dopamine receptors. Representative autoradiograms are shown for coronal sections of wild-type mouse brains incubated with radioligand for D1-type receptors (C) and D2-type receptors (D).

The significant increase in dopamine levels in triple mutant mice prompted us to measure levels of dopamine receptors in the striatum. We expected to see a compensatory change in dopamine receptor density in response to elevated dopamine levels in *Parkin* ^*-/-*^ *DJ-1* ^*-/-*^ *SOD1* ^*-/-*^ mice compared to wild type mice. Using radiolabeled dopamine receptor ligands, we quantified the abundance of D1-like and D2-like dopamine receptors in the striatum in fresh frozen sections. Contrary to the expected results, we saw no change in the binding of radiolabeled ligands to D1- or D2-type dopamine receptors (p > 0.4) (Figure 3). 

### Locomotor behavior is unchanged in mutant mice

**Figure 4:**
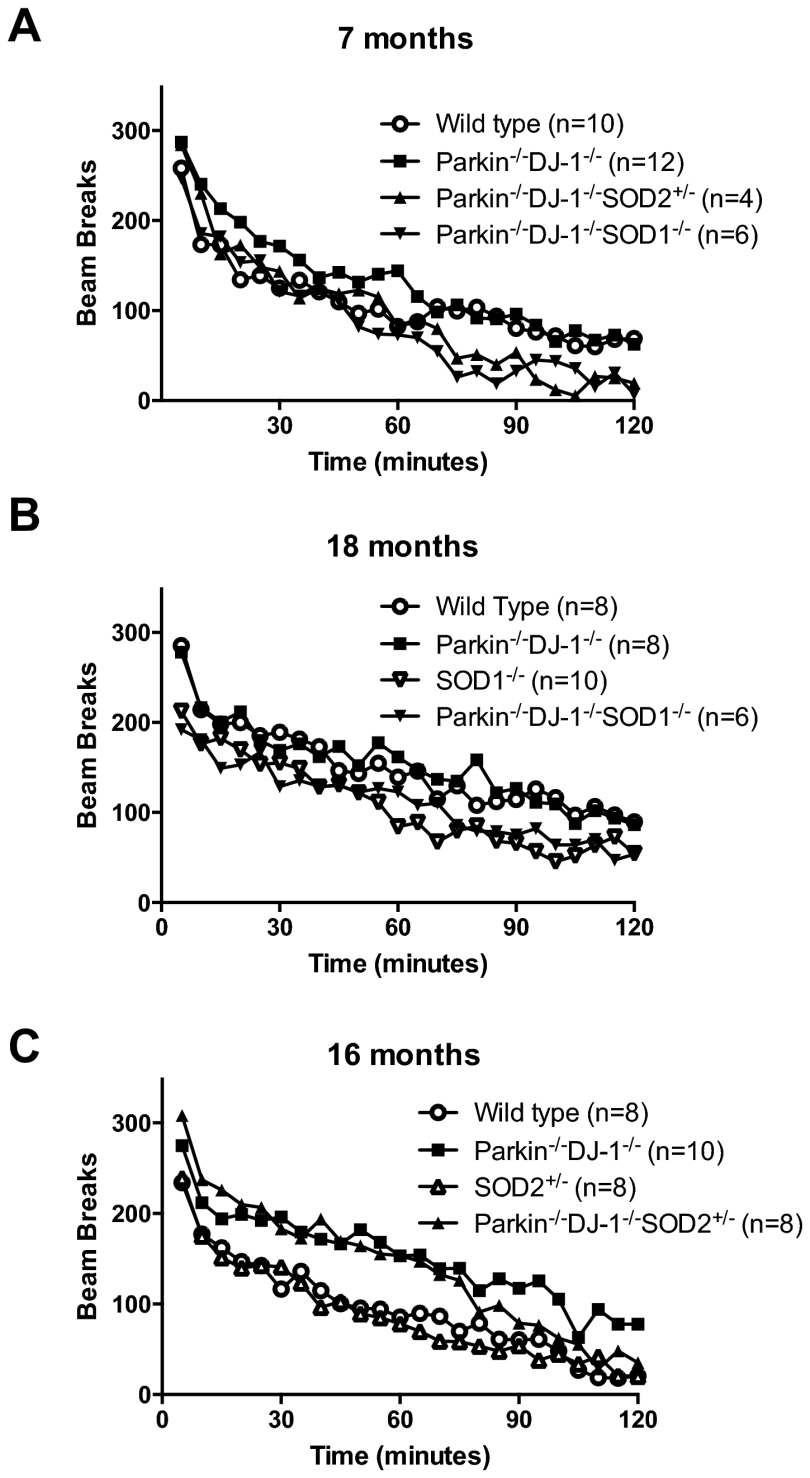
Locomotor behavior. Separate cohorts of mice were tested at 7 (A), 18 (B), and 16 (C) months. All cohorts of mice have an initial increase upon introduction to a new environment followed by decreased activity as the mice habituate. No significant differences are seen between genotypes in (A) and (B) by repeated measures ANOVA. There is a main effect of genotype in (C) (p = 0.005). Tukey post-hoc comparison shows a significant difference between Parkin^-/-^DJ-1^-/-^ mice and wild type (p = 0.031), and between Parkin^-/-^DJ-1^-/-^ and *SOD2* ^*+/-*^ (p = 0.015). For clarity, error bars are not shown.

Because Parkinson’s disease causes deficits in motor function, we investigated whether mice with combined PD-linked mutations have altered performance in established behavioral tests of motor function. The locomotor test measures total activity, and is used to identify mice that are hypoactive or hyperactive compared to wild type mice. Previous reports have indicated normal locomotor activity in *Parkin* ^*-/-*^ and *DJ-1* ^*-/-*^ mice [19,33,78]. We hypothesized that mice deficient for Parkin, DJ-1 and antioxidants would have an age-dependent locomotor deficit, manifested as reduced ambulatory behavior compared to age matched controls. The activity of all experimental groups was affected by time in the apparatus, indicating that all mice acclimated to the novel environment over the course of two hours, as indicated by a gradual decrease in the number of beam breaks. Seven-month old *Parkin* ^*-/-*^ *DJ-1* ^*-/-*^, *Parkin* ^*-/-*^ *DJ-1* ^*-/-*^ *SOD2* ^*+/-*^, and *Parkin* ^*-/-*^ *DJ-1* ^*-/-*^ *SOD1* ^*-/-*^ mice exhibited no locomotor deficits compared to wild type (p > 0.126) (Figure 4A). 

Analysis of locomotor activity in a 16-month old cohort revealed a significant difference between *Parkin* ^*-/-*^ *DJ-1* ^*-/-*^ mice and wild type (p = 0.031) and between *Parkin* ^*-/-*^ *DJ-1* ^*-/-*^ and *SOD2* ^*+/-*^ (p = 0.015) (Figure 4C), but not between *SOD2* ^*+/-*^ and wild type. 

### Rotarod behavior is altered in mice with combined mutations linked to PD

**Figure 5:**
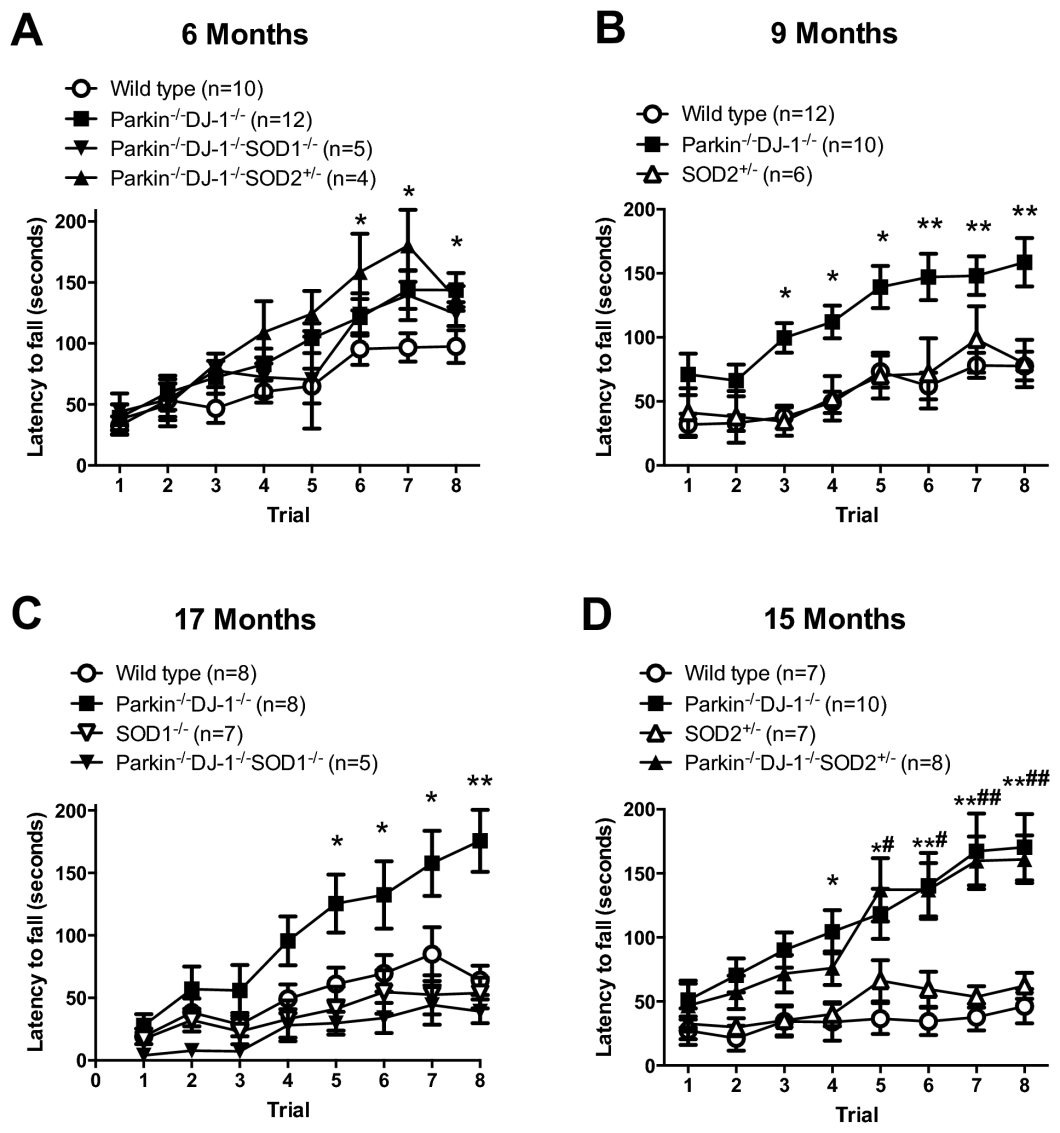
Parkin^-/-^DJ-1^-/-^ and aged Parkin^-/-^DJ-1^-/-^SOD2^+/-^ mice have improved rotarod performance. Latency to fall from an accelerating rotarod was measured for eight trials of five minutes over two days. Separate cohorts of mice were tested at 6 (A), 9 (B), 17 (C) and 15 (D) months. A minimum of 4 mice were tested per genotype per cohort. Symbols represent the mean ± SEM time (seconds) before falling off the rod for each of 8 trials. All genotypes learned the task over the course of multiple trials, however, Parkin^-/-^DJ-1^-/-^ mice had increased latency to fall compared to wild type. (*p<0.01, **p<0.001 by 2-way repeated measures ANOVA).

The rotorod task is commonly used to measure the ability of a rodent to sustain complex coordinated movement over time and has been used as a measure of basal ganglia function and as a functional measure of neurodegeneration in mouse models of amyotrophic lateral sclerosis (ALS), Huntington’s disease (HD) and other neurodegenerative diseases [79]. No abnormal rotorod phenotypes have been reported for *Parkin* ^*-/-*^, *DJ-1* ^*-/-*^, *SOD1* ^*-/-*^, or *SOD2* ^*+/-*^ mice, including results from our previously published studies [15,19,20,30,31,76,80,81]. We assessed the rotorod performance of wild type, *Parkin* ^*-/-*^ *DJ-1* ^*-/-*^, *SOD1* ^*-/-*^, *SOD2* ^*+/-*^, *Parkin* ^*-/-*^ *DJ-1* ^*-/-*^ *SOD1* ^*-/-*^, and *Parkin* ^*-/-*^ *DJ-1* ^*-/-*^ *SOD2* ^*+/-*^ at various ages (Figure 5). As expected, there was a main effect of trial for each experiment, indicating that there is a learning process associated with the task and all groups improve over the course of the 8 trials. There was also a main effect of genotype in each experiment and the *Parkin* ^*-/-*^ *DJ-1* ^*-/-*^ mice had an increased latency to fall instead of the expected decreased latency, compared to wild type mice. Although puzzling, this result confirms data that we previously reported on separate cohorts of *Parkin* ^*-/-*^ *DJ-1* ^*-/-*^ mice [76]. 

We first tested cohorts of 6-, 9-, 15-, and17-month old mice. Two-way repeated measures ANOVA showed that both number of trials (p < 0.001) and genotype (p <0.001) affected the latency to fall in all cohorts (Figure 5A-D). Posthoc analysis revealed a significant difference between wild type mice and *Parkin* ^*-/-*^ *DJ-1* ^*-/-*^ at all ages (p < 0.05) (Figure 5A-D), *Parkin* ^*-/-*^ *DJ-1* ^*-/-*^ *SOD2* ^*+/-*^ mice at 6 and 15 months (p <0.001) (Figure 5A, D), and *Parkin* ^*-/-*^ *DJ-1* ^*-/-*^ *SOD1* ^*-/-*^ mice at 6 and 17 months (p < 0.03) (Figure 5C). 

Rotarod performance is typically considered to be a measure of motor ability but other factors, such as weight, have also been reported to affect latency to fall [82]. We analyzed whether body weight of the mice in this study has an effect on rotarod performance by ANCOVA. There was no effect of body weight on latency to fall from the rotarod (p > 0.05).

**Figure 6:**
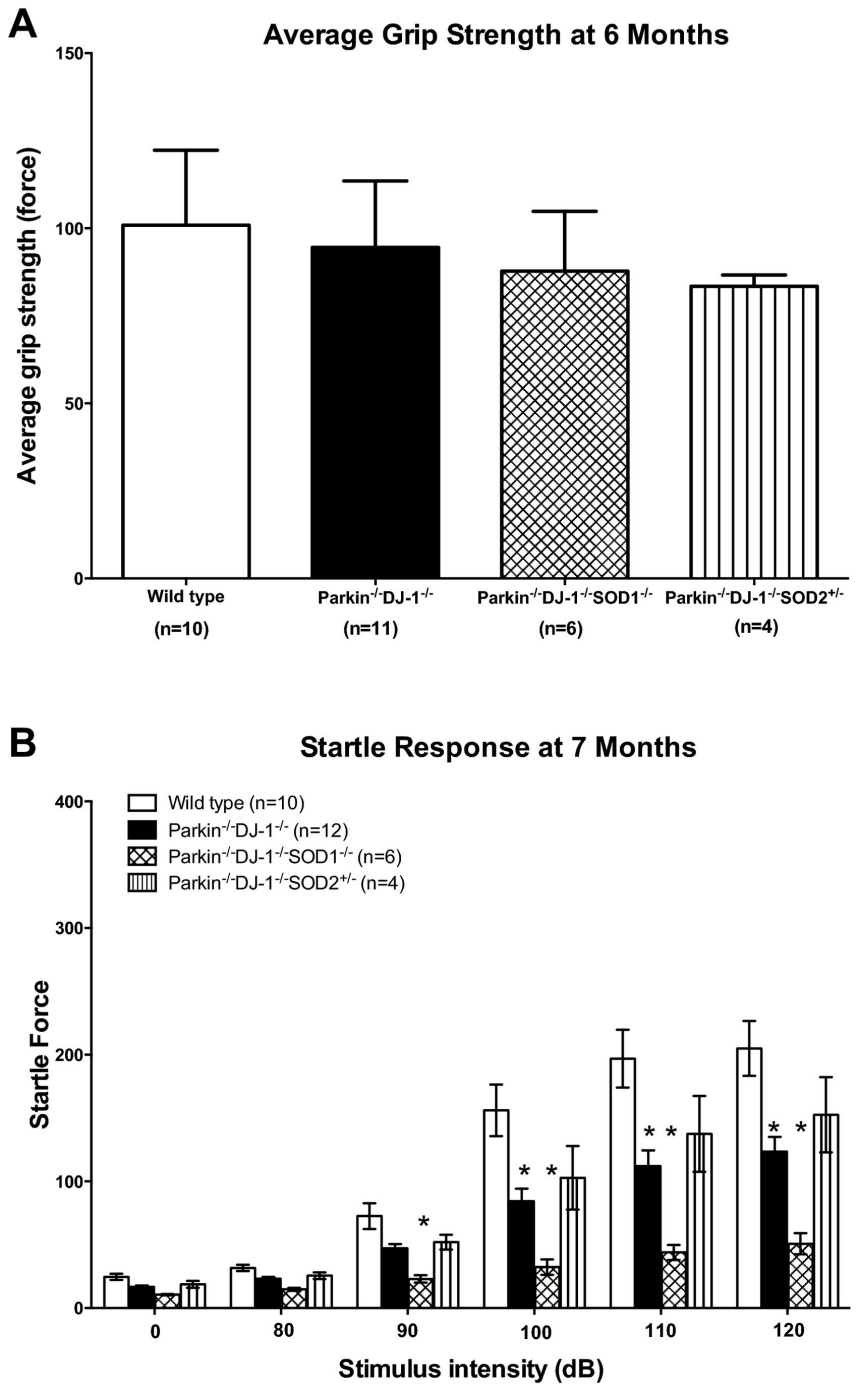
Knockout mice have similar grip strength and decreased startle response compared to wild type. (A) 6 month old Parkin^-/-^DJ-1^-/-^, Parkin^-/-^DJ-1^-/-^SOD1^-/-^ and Parkin^-/-^DJ-1^-/-^SOD2^+/-^ mice had average grip strength statistically similar to wild type mice by 1 way ANOVA (p = 0.07). (B) The intensity of the response to an audible stimulus was measured in a cohort of 7 month old mice (n = 4-12 mice per genotype). (*p < 0.0001 by 2 way ANOVA).

The increase in rotorod performance of *Parkin* ^*-/-*^ *DJ-1* ^*-/-*^ mice prompted us to determine whether these mice have increased ability to grip the rotating rod, thus facilitating their ability to remain on the rod for longer periods of time. We analyzed the grip strength of a cohort of 6-month old wild type, *Parkin* ^*-/-*^ *DJ-1* ^*-/-*^, *Parkin* ^*-/-*^ *DJ-1* ^*-/-*^ *SOD1* ^*-/-*^, and *Parkin* ^*-/-*^ *DJ-1* ^*-/-*^ *SOD2* ^*+/-*^ mice and found no difference between genotypes (p=0.352) (Figure 6A). Therefore the improved rotorod performance of the double and triple mutant mice was not due to increased grip strength. 

Other characteristics that could underlie the striking differences observed in rotarod behavior are differences in reflexes or sensory functions. This prompted us to measure startle response behavior in the mice using the acoustic startle test, which measures the innate reflex of the mouse in response to a sudden and unexpected acute stimulus, such as a loud noise. Analysis of a cohort of 7 month old wild type, *Parkin* ^*-/-*^ *DJ-1* ^*-/-*^, *Parkin* ^*-/-*^ *DJ-1* ^*-/-*^ *SOD1* ^*-/-*^, and *Parkin* ^*-/-*^ *DJ-1* ^*-/-*^ *SOD2* ^*+/-*^ mice, showed a significant decrease in startle response in *Parkin* ^*-/-*^ *DJ-1* ^*-/-*^ and *Parkin* ^*-/-*^ *DJ-1* ^*-/-*^ *SOD1* ^*-/-*^ (p < 0.001) compared to wild type (Figure 6B). There was also a trend toward reduced startle response in *Parkin* ^*-/-*^ *DJ-1* ^*-/-*^ *SOD2* ^*+/-*^ mice compared to wild type mice, but this was not significant, perhaps due to the smaller sample size of this genotype. Decreased startle responses in *Parkin* ^*-/-*^ *DJ-1* ^*-/-*^ and *Parkin* ^*-/-*^ *DJ-1* ^*-/-*^ *SOD1* ^*-/-*^ indicate impairment of reflexes and/or sensory functions compared to wild type mice. However, this would not explain the observed increased latency to fall in these genotypes.

Because decreased startle amplitudes could indicate decreased anxiety levels and because fear of falling off the rotarod apparatus could contribute to rotarod behavior performance, we tested wild type and mutant mice for changes in fear or anxiety using several established behavioral paradigms including the light/dark test, the open field test and the elevated plus maze. We did not observe any difference between genotypes in any of these behavior tests (data not shown). Therefore, the increased rotorod performance of the double and triple mutant mice does not appear to be due to altered anxiety.

**Figure 7:**
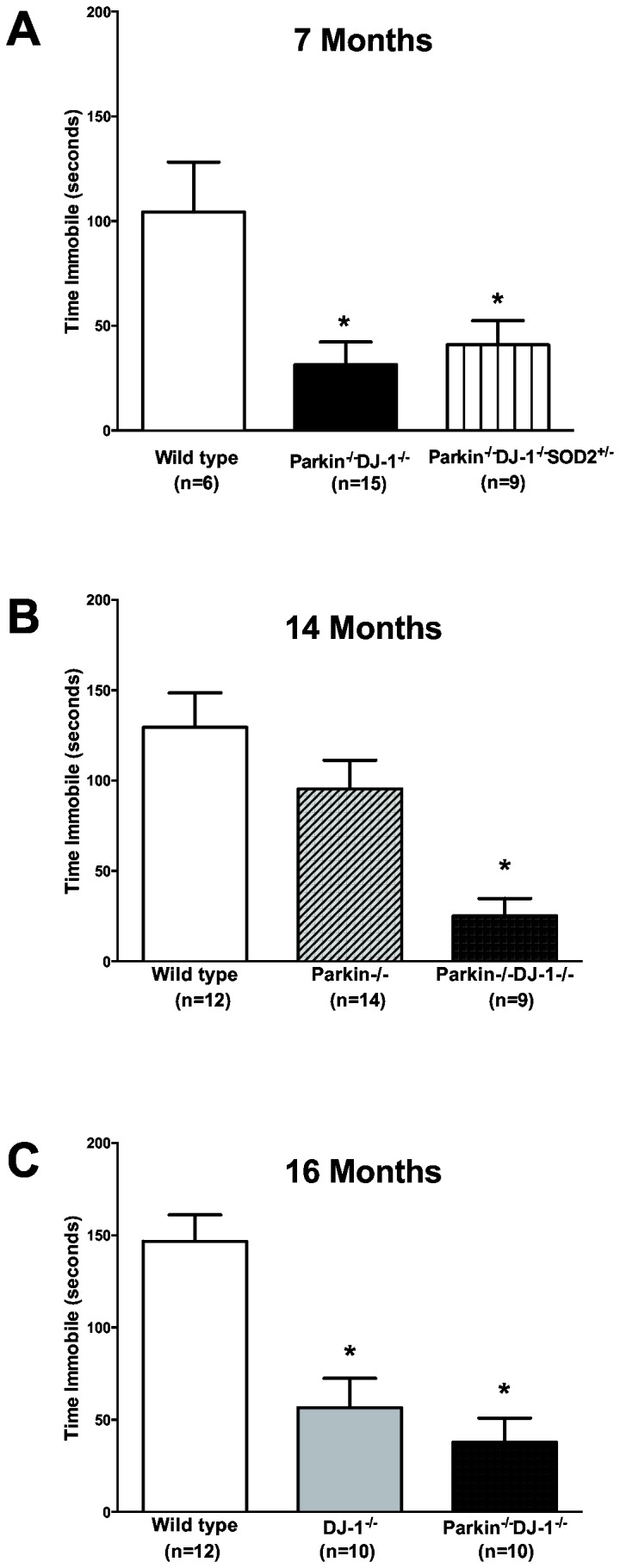
DJ-1^-/-^, Parkin^-/-^DJ-1^-/-^ and Parkin^-/-^DJ-1^-/-^SOD2^+/-^ mice have decreased time immobile in the Porsolt forced swim test. Separate cohorts of mice were tested at age 6 months with a minimum n = 6. (*p ≤ 0.01 by 1-way ANOVA).

Finally, we conducted the forced swim test as part of our comprehensive battery of behavioral tests in an effort to better characterize these mice and possibly explain the altered rotarod performance. The forced swim test (FST) is a test of forced motor behavior developed to predict the efficacy of antidepressant drugs and is particularly sensitive to compounds acting on the serotonin and norepinephrine systems. Compared to wild type, we found significantly reduced immobility time in 6-, 14- and 16-month old *Parkin* ^*-/-*^ *DJ-1* ^*-/-*^ mice (p = 0.005), 6-month old *Parkin* ^*-/-*^ *DJ-1* ^*-/-*^ *SOD2* ^*+/-*^ mice (p = 0.027) and 16-month old *DJ-1* ^*-/-*^ mice (p < 0.001) (Figure 7A-C). Post hoc analysis of a second 14 month old cohort of wild type, *Parkin* ^*-/-*^, and *Parkin* ^*-/-*^DJ-1^- /-^ demonstrated a significant decrease in immobility time *Parkin* ^*-/-*^ *DJ-1* ^*-/-*^ mice compared to wild type (p < 0.001). Together these data indicate that the loss of DJ-1 alone is sufficient to confer the increased mobility observed in the forced swim test.

### Normal muscle, heart, and cerebellar histology in Parkin^-/-^DJ-1^-/-^ *mice*

**Figure 8:**
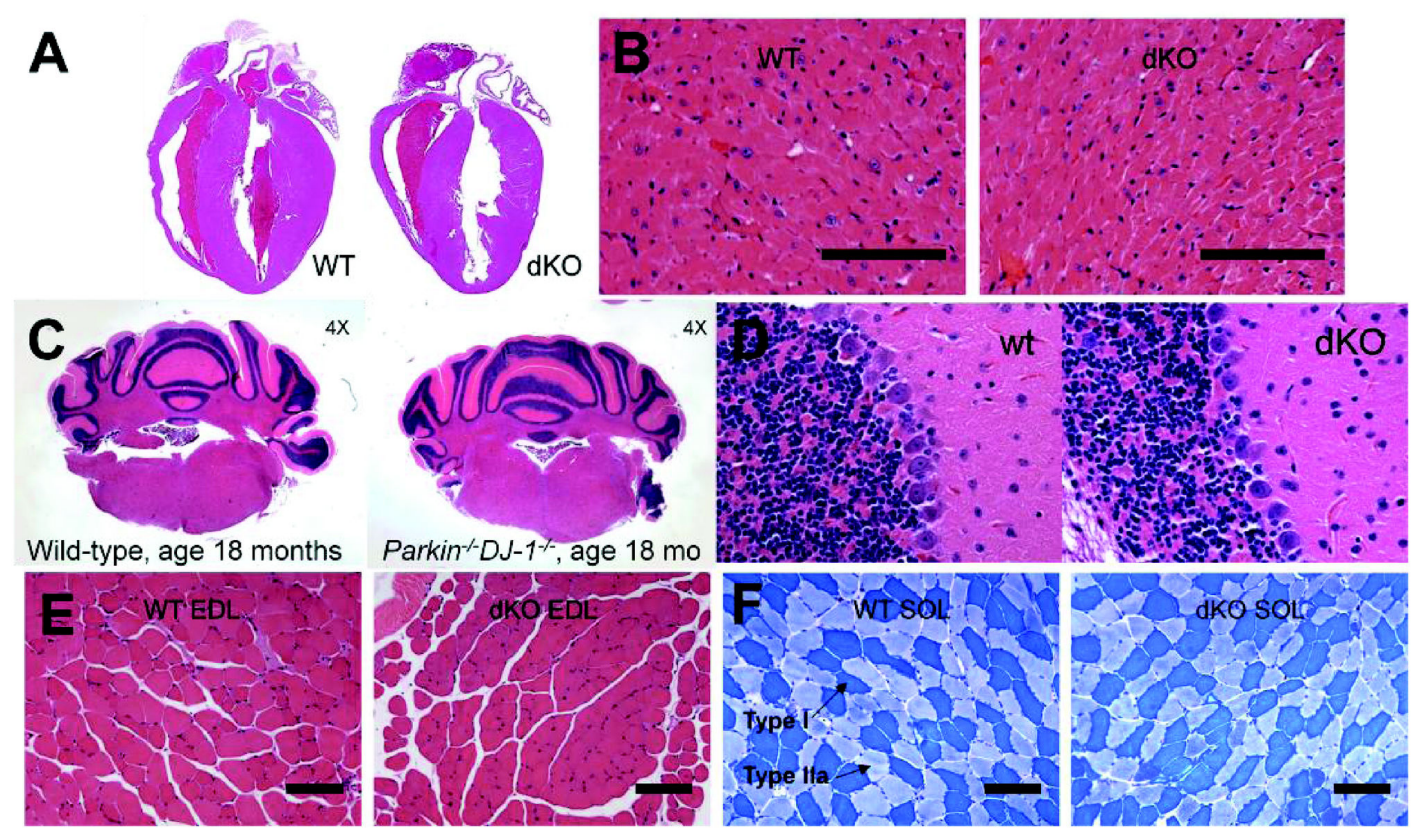
Muscle, heart and cerebellar histology of Parkin^-/-^DJ-1^-/-^ mice is not different from wild type mice. Hematoxylin and eosin staining of sections of the heart (A, B), the cerebellum (C, D) and the extensor digitorum longus (EDL) muscle (E) appeared normal in Parkin^-/-^DJ-1^-/-^ (dKO) mice compared to wild type mice. Metachromatic ATPase staining (F) showed comparable distribution of dark blue Type I fibers and lighter blue Type II fibers in soleus muscle from wild type and *Parkin* ^*-/-*^DJ-1^-/-^ mice. Scale bar = 100 μm.

Parkin is highly expressed in the heart and muscle tissue, as well as most areas of the brain [7]. Additionally, the ability to stay on the rotorod is highly dependent on cerebellar function. Therefore, histology of muscle, heart and cerebellum was performed in order to test for abnormalities that might account for enhanced rotarod performance in the *Parkin* ^*-/-*^ *DJ-1* ^*-/-*^ mice. Hematoxylin and eosin staining of paraffin sections of hearts from 18 month old wild type and *Parkin* ^*-/-*^ *DJ-1* ^*-/-*^ mice showed normal organ structure as well as normal cellular appearance of heart muscle in mice of both genotypes (Figure 8). Hematoxylin and eosin staining of coronal brain sections showed normal anatomical and cellular organization of the cerebellum (Figure 8). We hypothesized we would see a difference in the ratio of fast twitch to slow twitch muscle fiber types, providing the *Parkin* ^*-/-*^ *DJ-1* ^*-/-*^ mice fatigue-resistant muscles and better stamina on the rotorod. The ratio and morphology of type I and type II muscle fiber types appeared similar in metachromatic ATPase stained soleus muscle from *Parkin* ^*-/-*^ *DJ-1* ^*-/-*^ mice compared to wild type mice (Figure 8). 

## Discussion

Despite years of effort by many researchers generating and characterizing mice with recessive mutations in Parkin and DJ-1 linked to familial PD, no Parkin or DJ-1 knockout mouse line exhibits all, or even most, of the key clinical and neuropathological features of PD [14,16,17,19-27,30,31]. This contrasts with mouse models of other neurodegenerative diseases such as Huntington’s disease and amyotrophic lateral sclerosis that reproduce the major neuropathological and behavioral features of these diseases, including delayed onset of motor behavior deficits as well as selective and progressive neurodegeneration and protein inclusions [83,84]. It is possible that compensatory changes protect mice from the neurodegeneration caused by Parkin and DJ-1 mutations or that additional stress is required to initiate neurodegeneration in Parkin and DJ-1 knockout mice. Indeed, we have shown that Parkin knockout mice are more susceptible to nigral cell loss induced by chronic lipopolysaccharide exposure [18] and others have shown that DJ-1 knockout mice are more susceptible to MPTP-induced nigral cell loss [22]. Here we sought to generate better mouse models of progressive nigral cell loss induced only by genetic mutations. We combined PD-linked mutations in Parkin and DJ-1 with deficiency for antioxidant enzymes that have been shown to be important determinants of susceptibility to nigrostriatal degeneration in mice [59-64,66-72,85,86].

Because *SOD1* ^*-/-*^ mice and *SOD2* ^*+/-*^ mice have been shown to be more susceptible to neurotoxin-mediated nigral cell loss [60-65,68-73,86-88] we hypothesized that combining Parkin and DJ-1 deficiency with SOD1 or SOD2 deficiency would cause a loss of dopaminergic neurons in the substantia nigra. Contrary to our expectations, neither *Parkin* ^*-/-*^ *DJ-1* ^*-/-*^ *SOD1* ^*-/-*^ mice nor *Parkin* ^*-/-*^ *DJ-1* ^*-/-*^ *SOD2* ^*+/-*^ mice showed age-dependent loss of nigral dopamine neurons compared to wild type mice (Figure 1). It has been suggested that death of nigral dopamine neurons begins with the axon terminals in the striatum, from which a die-back process occurs, ultimately resulting in the loss of nigral neurons after loss of terminals has occurred [87,89,90]. For this reason, we also measured the levels of dopamine and its metabolites in the striatum (Figure 2). Remarkably, *Parkin* ^*-/-*^ *DJ-1* ^*-/-*^ *SOD1* ^*-/-*^ mice but not *Parkin* ^*-/-*^ *DJ-1* ^*-/-*^ mice or *SOD1* ^*-/-*^ mice had significantly increased levels of dopamine in the striatum. This indicates that SOD1 deficiency perturbs the nigrostriatal pathway, but only in combination with mutations linked to PD. The increased striatal dopamine in *Parkin* ^*-/-*^ *DJ-1* ^*-/-*^ *SOD1* ^*-/-*^ mice is consistent with the increased striatal dopamine we identified in mice deficient for Parkin, DJ-1 and another antioxidant protein, glutathione peroxidase 1 [76] and consistent with previously published results indicating an increase in striatal dopamine in aged *Parkin* ^*-/-*^ *DJ-1* ^*-/-*^ *PINK1* ^*-/-*^ mice [23]. Additionally, our data agrees with studies indicating that loss of *Parkin* alone [31,91] and loss of *DJ-1* alone does not alter dopamine metabolism [20,22,24,78], but contrasts with reports indicating moderate changes in dopamine turnover in *Parkin* and *DJ-1* single knockout mice [21,33,92]. The lack of a significant change in striatal dopamine levels in *Parkin* ^*-/-*^ *DJ-1* ^*-/-*^ *SOD2* ^*+/-*^ mice may be due to SOD2 heterozygosity not being sufficient to produce a full phenotype in these mice. SOD2 homozygous knockout mice are not viable, thus precluding analysis of *Parkin* ^*-/-*^ *DJ-1* ^*-/-*^ *SOD2* ^*-/-*^ mice.

The increased striatal dopamine did not cause hyperactivity or other apparent behavioral abnormalities specific to *Parkin* ^*-/-*^ *DJ-1* ^*-/-*^ *SOD1* ^*-/-*^ mice. Dopamine levels are tightly regulated by presynaptic autoreceptors that signal the regulation of tyrosine hydroxylase to alter dopamine synthesis. Therefore, we speculate that the increased striatal dopamine levels indicate that *Parkin* ^*-/-*^ *DJ-1* ^*-/-*^ *SOD1* ^*-/-*^ mice either have dopamine autoreceptor signaling defects or that the increased dopamine compensates for other defects in dopaminergic signaling. It is possible that, in the absence of the compensatory increase in striatal dopamine, we would have detected behavioral phenotypes specifically in *Parkin* ^*-/-*^ *DJ-1* ^*-/-*^ *SOD1* ^*-/-*^ mice. Alternatively, the increase in dopamine may be indicative of a compensatory change that is occurring in the striatum that might ultimately lead to neuronal death, either due to overwhelming the compensatory mechanisms of the cell or due to neurotoxicity of dopamine itself [93,94]. The increased striatal dopamine also could be due to interactions between the three genes to affect release, trafficking, dopamine production, or degradation. Consistent with this possibility, Parkin and DJ-1 have been found in cell membranes and vesicles [95,96] and mutations in Parkin and DJ-1 can cause altered dopamine release, reuptake and signaling [20,97,98]. 

The increased striatal dopamine levels cannot explain the rotarod and forced swim test behavioral phenotypes because we did not observe an increase in striatal dopamine in the DJ-1 single knockout mice (which show the forced swim test phenotype) or Parkin-DJ-1 double knockout mice (which show the improved rotarod performance phenotype). Therefore, the behavioral and the neurochemical phenotypes observed in these mice are likely independent of each other. Mice deficient for both Parkin and DJ-1 consistently stayed on the rotarod longer than wild type mice. This finding holds true in both the young and aged cohorts of mice, although the phenotype is more pronounced in aged mice (Figure 6). The rotarod behavioral phenotype is consistent with independent cohorts of *Parkin* ^*-/-*^DJ-1^-/-^ mice that we studied in combination with glutathione peroxidase deficiency [76], which suggested that the increased rotarod latency of *Parkin* ^*-/-*^ *DJ-1* ^*-/-*^ mice may be due to decreased distraction from the task compared to wild type mice. 

The decreased startle response in *Parkin* ^*-/-*^ *DJ-1* ^*-/-*^ mice might be due to Parkin deficiency alone because *Parkin* ^*-/-*^ mice have previously been shown to have decreased startle response [31]. The greater decrease in startle response in *Parkin* ^*-/-*^ *DJ-1* ^*-/-*^ *SOD1* ^*-/-*^ mice is likely due to a loss of hearing in mice deficient for SOD1. The cochleae of *SOD1* ^*-/-*^ mice have been shown to have severe spiral ganglion cell degeneration by 7 months of age [99]. 

It remains possible that abnormalities in antioxidants such as SOD1 or SOD2 are involved in the development or progression of PD. However, our study demonstrates that deficiency for these antioxidants does not directly cause nigral cell loss or parkinsonian-like locomotor deficits in the context of mice with PD-linked mutations in Parkin and DJ-1. The increase in striatal dopamine resulting from the combination of SOD1, Parkin and DJ-1 deficiency might represent early-stage nigrostriatal dysfunction and these mice may be useful for efforts to develop neuroprotective therapies targeting early stage abnormalities in PD pathogenesis.
