# Correction: Behavioral and Neurotransmitter Abnormalities in Mice Deficient for Parkin, DJ-1 and Superoxide Dismutase

**DOI:** 10.1371/journal.pone.0091128

**Published:** 2014-02-27

**Authors:** The PLOS ONE Staff

Figure 1 and Figure 8 are updated here for better readability.

**Figure 1:**
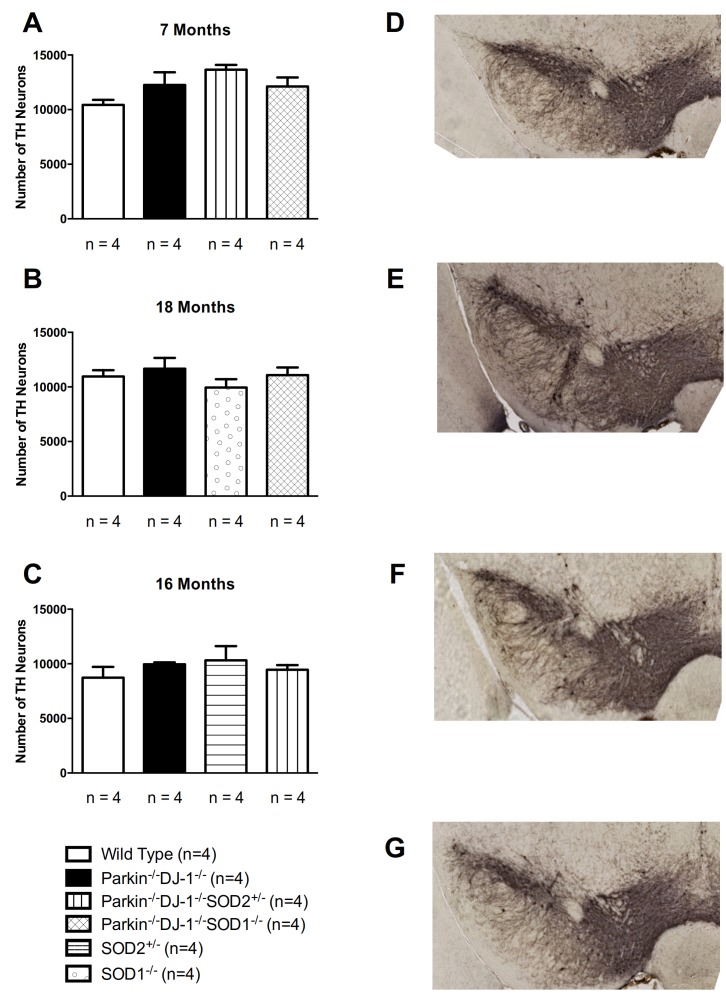
No changes in the total bilateral number of TH-positive nigral neurons estimated by rigorous stereology. Dopaminergic neurons of separate cohorts of mice were counted for 7 month old mice (A) and 18 month old mice (B) and 16 month old mice (C). n  =  4 for all genotypes. One-way ANOVA showed no differences between any of the genotypes (p ≥ 0.1). Representative images of TH-stained coronal sections used for analysis are shown for 7 month old wild-type (D), Parkin-/-DJ-1-/- (E), Parkin-/-DJ-1-/-SOD2+/- (F) and Parkin-/-DJ-1-/-SOD1-/- (G).

**Figure 8:**
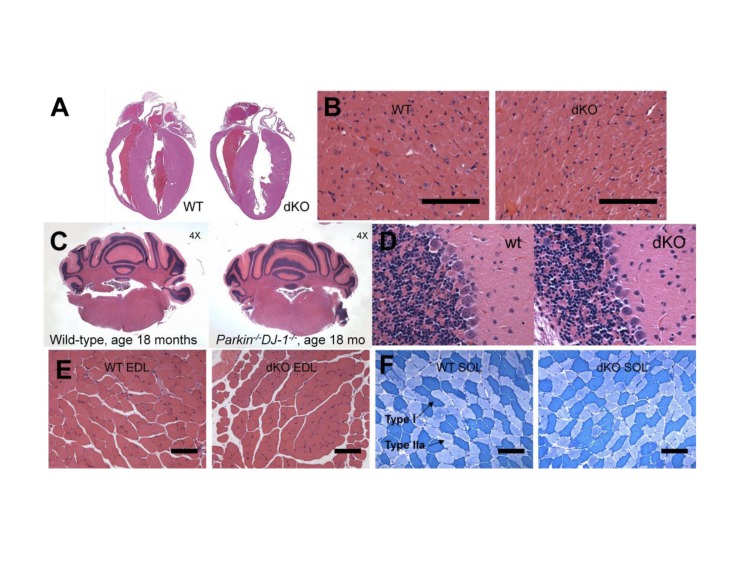
Muscle, heart and cerebellar histology of Parkin-/-DJ-1-/- mice is not different from wild type mice. Hematoxylin and eosin staining of sections of the heart (A, B), the cerebellum (C, D) and the extensor digitorum longus (EDL) muscle (E) appeared normal in Parkin-/-DJ-1-/- (dKO) mice compared to wild type mice. Metachromatic ATPase staining (F) showed comparable distribution of dark blue Type I fibers and lighter blue Type II fibers in soleus muscle from wild type and Parkin-/-DJ-1-/- mice. Scale bar  =  100 μm.
